# Editorial: New marketable discoveries in nanobiotechnology

**DOI:** 10.3389/fbioe.2024.1412587

**Published:** 2024-04-19

**Authors:** Ronnie G. Willaert, Sandor Kasas

**Affiliations:** ^1^ Research Group Structural Biology Brussels, Alliance Research Group VUB-UGhent NanoMicrobiology (NAMI), Vrije Universiteit Brussel (VUB), Brussels, Belgium; ^2^ International Joint Research Group VUB-EPFL BioNanotechnology & NanoMedicine (NANO), Brussels, Switzerland; ^3^ Laboratory of Biological Electron Microscopy, Ecole Polytechnique Fédérale de Lausanne (EPFL) and University of Lausanne, Lausanne, Switzerland; ^4^ Centre Universitaire Romand de Médecine Légale, UFAM, Université de Lausanne, Lausanne, Switzerland

**Keywords:** orthopedic surgery, nanochitosan encapsulation, 3D printing, fomatidine nanoparticles, nanomotion detection, thryroid-stimulating hormone assay, cardiac tissue contractile force, machine-learning (ML)

With the potential to advance the current scientific landscape, nanobiotechnology represents a flourishing area of research worldwide. With the increasingly innovative approaches being worked on by scientists, we foresee that this will significantly contribute to next-generation development and application in this field. The Research Topic aims to collect and disseminate new marketable discoveries in nanobiotechnology science worldwide to a global audience, promoting its development with the evolutionary concepts and ideas from laboratories across the globe.

Deng et al. reviewed the present state of nanotechnology in orthopedic surgery, its applications as well as possible future developments. In recent years, numerous medical fields have been revolutionized by the advent of nanotechnology. The authors reviewed comprehensively the recent developments of nanotechnology in the field of orthopedic surgery. More specifically these discussed the impact of nanotechnology in joint replacements, drug delivery systems, orthopedic oncology, chondral and osseous deformities, implant material, as well as applications for imaging and the developments of novel sensors for diagnosis.

Freitas et al. evaluated the antimicrobial and wound-healing potential of naphthoquinones encapsulated in nanochitosan. The use of chitosan to encapsulate pharmacologically active compounds has many advantages such as its biocompatibility, biodegradability, and low cost. The coumpounds naphthoquinones 3- chloromethylene-menadione (NQ1) and 2,3-dichloro-1,4-naphthoquinone (NQ2) were nanoencapsulated into chitosan (CNP) by the ionotropic gelatinization technique and evaluated for their antimicrobial and wound healing activities. Both encapsulated compounds retained their naphthoquinone antimicrobial activity against *Staphylococcus aureus*, *Staphylococcus epidermidis*, *Streptococcus pyogenes* and *Pseudomonas aeruginosa*. Only CNP-NQ1 displayed low toxicity for safe use in human tissues. Scratch assays suggested successfully wound healing properties of the compound that prevent bacterial contamination, and accelerate dermal tissue recovery.

Alogla reviewed how antioxidant delivery for therapeutic strategies can be enhanced by using 3D printing. Incorporating antioxidants into 3D-printed matrices enables controlled release and localized delivery, improving efficacy while minimizing side effects. Encapsulation techniques such as direct mixing, coating, and encapsulation are discussed. The author also reviewed various 3D printing methods and materials, printing parameters, as well as post-processing methods to influence antioxidant release kinetics and stability. Applications include wound healing, tissue regeneration, 3D-printed bone scaffolds, drug delivery, and foods with 3D-printed antioxidants.

Nagai et al. developed orally disintegrating tablets containing famotidine nanoparticles that provide high intestinal absorbability. Famotidine (FAM) nanoparticles were used to enhance oral bioavailability. Dispersions containing FAM nanoparticles with a particle size of approximately 50–220 nm were produced by the bead-milling treatment and included in an orally disintegrating tablet formulation. *In vivo* evaluation in rats showed improved mucosal permeability and oral bioavailability.

Kweku et al. developed a machine-learning method for the classification of the state of living organisms’ oscillations as detected by Atomic Force Microscopy (AFM)-based nanomotion detectors. Such devices provide rapid antibiotic sensitivity tests that are crucial for the treatment of bacterial infections. The interpretation of the data delivered by such devices is not trivial and in this contribution, the authors implemented a machine-learning algorithm that classifies bacterial responses to antibiotics. The efficiency of the method was demonstrated by classifying living *Bordetella pertussis* bacteria in the virulent or avirulence phase, and dead bacteria, based on their cellular nanomotion signal. The method offers significant advantages over current approaches, as it is significantly faster and more accurate.

Arai et al. developed a thyroid-stimulating hormone assay system for a benchtop digital ELISA desktop analyzer. Regular checkups for thyroid-stimulating hormone (TSH) levels are essential for the diagnosis of thyroid disease. By utilizing the ultrasensitivity of the digital immunoassay (d-IA) based on functionalized magnetic beads, the authors were able to use a sample volume of as little as 5 µL for each assay. The correlation between serum and plasma levels indicated a good agreement and a sensitivity of 0.002280 μIU/mL was obtained.

Ma et al. developed a high-throughput screening system based on micropillars for three-dimensional engineered cardiac tissues (3D ECTs). To balance the inherent tradeoff among imaging resolution, field of view, and speed, an innovative mosaic imaging system was designed, built, and validated to sense the contractile force of 3D ECTs in a 96-well plate setup. The system performance was validated for up to 3 weeks. The developed method significantly reduces the cost, time, and labor needed for preclinical cardiotoxicity assay.

